# Unraveling the genetic diversity of *Ceiba pubiflora* (Malvaceae) in isolated limestone outcrops: Conservation strategies

**DOI:** 10.1371/journal.pone.0299361

**Published:** 2024-04-01

**Authors:** Murilo Malveira Brandão, Fábio de Almeida Vieira, Abidã Gênesis da Silva Neves, Rubens Manoel dos Santos, Dulcineia de Carvalho, Elytania Veiga Menezes, Patrícia Abreu de Moreira, Dario Alves de Oliveira, Afrânio Farias de Melo Júnior, Vanessa de Andrade Royo

**Affiliations:** 1 Department of Biological Sciences, State University of Montes Claros, Montes Claros, Minas Gerais, Brazil; 2 Academic Unit Specialized in Agricultural Sciences, Federal University of Rio Grande do Norte, Macaíba, Rio Grande do Norte, Brazil; 3 Department of Forest Science, Federal University of Lavras, Lavras, Minas Gerais, Brazil; 4 Institute of Exact and Biological Sciences, Federal University of Ouro Preto, Ouro Preto, Minas Gerais, Brazil; Nuclear Science and Technology Research Institute, ISLAMIC REPUBLIC OF IRAN

## Abstract

Seasonally Dry Tropical Forests (SDTFs) located on limestone outcrops are vulnerable to degradation caused by timber logging and limestone extraction for cement production. Some of these forests represent the last remnants of native vegetation cover, functioning as isolated islands. *Ceiba pubiflora* (Malvaceae) is a tree frequently found on limestone outcrops in the central region of Brazil. This study aimed to evaluate the genetic diversity and identify suitable populations for the establishment of Management Units (MUs) for conservation. Inter-simple sequence repeat markers were employed to assess the genetic diversity in ten populations sampled from the Caatinga, Cerrado, and Atlantic Forest biomes. The species exhibited substantial genetic diversity (H_T_ = 0.345; P_LP_ = 97.89%). Populations SAH, JAN, and MON demonstrated elevated rates of polymorphic loci (> 84.2%) along with notable genetic diversity (He > 0.325). Additionally, these populations were the primary contributors to gene flow. The analysis of molecular variance (AMOVA) indicated that most genetic variation occurs within populations (91.5%) than between them. In the Bayesian analysis, the ten populations were clustered into five groups, revealing the presence of at least three barriers to gene flow in the landscape: 1) the Central Plateau or Paranã River valley; 2) near the Espinhaço mountains or the São Francisco River valley; and 3) around the Mantiqueira mountain range, Chapada dos Veadeiros plateau, and disturbed areas. A positive and statistically significant correlation was observed between genetic (θ^B^) and geographic distances (r = 0.425, p = 0.008). Based on these findings, we propose the establishment of Management Units in Minas Gerais state, encompassing the (1) southern region (MIN population), (2) central region (SAH population), and (3) north region (MON population), as well as in Goiás state, covering the (4) Central Plateau region. These units can significantly contribute to preserving the genetic diversity of these trees and protecting their habitat against ongoing threats.

## Introduction

Seasonal Dry Tropical Forests (SDTFs) are highly vulnerable to degradation due to agricultural expansion and urban development. These ecosystems possess distinctive historical characteristics, specialized soil and climate conditions, and unique phytosociological features [1, 2]. SDTFs are defined as ecosystems found in tropical regions characterized by a predominance of seasonal rains and pronounced dry periods [3]. During the dry season, the vegetation experiences significant leaf loss, often exceeding 50% [4]. SDTFs are characterized by soils with high fertility and moderately high pH, occurring in regions that receive seasonal rainfall of less than 1,600 mm per year and experience at least five months of dry season [5, 6].

Currently, the distribution of SDTFs in South America is discontinuous, ranging from the Caatinga in the Brazilian Northeast to the Uruguay River valley, with smaller areas extending into the Amazon Basin and towards Mexico. Prado and Gibbs et al. [7] identified three main centers: the ‘Caatingas nucleus’ in Northeast Brazil, the ‘Misiones nucleus’ along the Paraguay-Paraná River system, and the ‘Piedmont nucleus’ in Southwestern Bolivia and Northwestern Argentina. Smaller and isolated clusters of SDTFs are also found in dry valleys across the Andes, spanning Bolivia, Northern Peru, and Central Brazil. These clusters occur as enclaves, predominantly on limestone outcrops, within various biome physiognomies. It is hypothesized that these forests represented a continuous and interconnected distribution during the dry period of the Pleistocene, approximately 21,000 years ago [7, 8].

Limestone outcrops in seasonal areas of Brazil promote the occurrence of unique forest formations. Within these environments, vegetation species adapted for drought resistance are observed, including some species restricted to other rocky habitats and occurring in more mesic environments and different of plant formations [2]. In Central Brazil, limestone outcrops harbor forests that can be considered centers of diversity [9], sheltering species adapted to climatic stress and unique soil conditions. Limestone outcrops play a crucial role as habitat patches for various species of SDTFs and serve as repositories of genetic diversity in many areas [2, 10]. Moreover, in certain areas, forests on limestone outcrops represent the last vestiges of native vegetation cover, standing as isolated islands amidst an array of grazing or agricultural lands [11].

Maintaining the genetic diversity of tree species populations in these landscapes is crucial for long-term survival. SDTFs on limestone outcrops require special attention as they face threats from limestone exploitation for cement plants, timber harvesting, and agricultural expansion. Specifically, studies focusing on the genetic diversity of populations in these remnants are essential to identify areas suitable for designation as conservation units, employing landscape genetics [12]. Landscape genetics is an interdisciplinary field of research that has emerged in this century, integrating population genetics, landscape ecology, and spatial statistics [12–15]. The insights gained from landscape genetics are essential for proposing the establishment of conservation units and optimizing seed collection to sample the genetic variability of the species efficiently. Identifying these areas is crucial for preserving populations capable of maintaining the evolutionary heritage of the species, known as Evolutionarily Significant Units (ESUs) [16–18].

Seasonal deciduous forests on outcrops are environments that are still relatively well-preserved [19]. These areas contribute to the maintenance of long-term population viability and genetic diversity [10], including a diversified community [20, 21]. Additionally, certain trees such as *Acrocomia aculeata* [22], *Astronium urundeuva* [23], *Caryocar coriaceume* [24] and *Tabebuia roseoalba* [25] have demonstrated high genetic diversity in similar physiognomies. Among the various tree species in the SDTFs on limestone outcrops in Central Brazil, *Ceiba pubiflora* (A.St.-Hil.) K. Schum (Malvaceae) stands out. The geographic distribution of this species primarily extends across Central Brazil but also includes some regions in Paraguay and less is frequent in Argentina [26]. This species produces flowers during the dry season, typically between June to August. Bees and hummingbirds often visit these flowers. The seeds of *Ceiba pubiflora* are characterized by their lightweight and are surrounded by hairy arils, which facilitate their anemochory [26]. While some molecular studies have been conducted with this species [27], this is the first populational study to focus on its most significant occurrence region. This region has been significantly impacted by anthropogenic activities, particularly limestone extraction, leading to genetic erosion in species characteristic of these environments [11].

Thus, population genetics studies can indicate important aspects of conservation biology, such as the loss of genetic diversity in small and isolated populations [28, 29]. *Ceiba pubiflora* was chosen for this study due to its frequent occurrence in limestone outcrops of Central Brazil. It is a model to verify patterns of genetic differentiation between populations and regions. Therefore, the aim of this study was to assess the genetic diversity within natural populations of *Ceiba pubiflora*, using Inter simple sequence repeat (ISSR) markers, a cost-effective and straightforward method for efficiently analyzing the genetic diversity across numerous polymorphic bands [30–32]. We test the following hypotheses: (1) populations of *Ceiba pubiflora*, in natural fragments on limestone outcrops, retain high genetic diversity; (2) the genetic and geographic distances among populations are positively correlated, indicating isolation by distance; (3) the genetic differentiation among populations is influenced by geographic barriers.

## Materials and methods

### Characterization of populations and sampling

**Fig 1:**
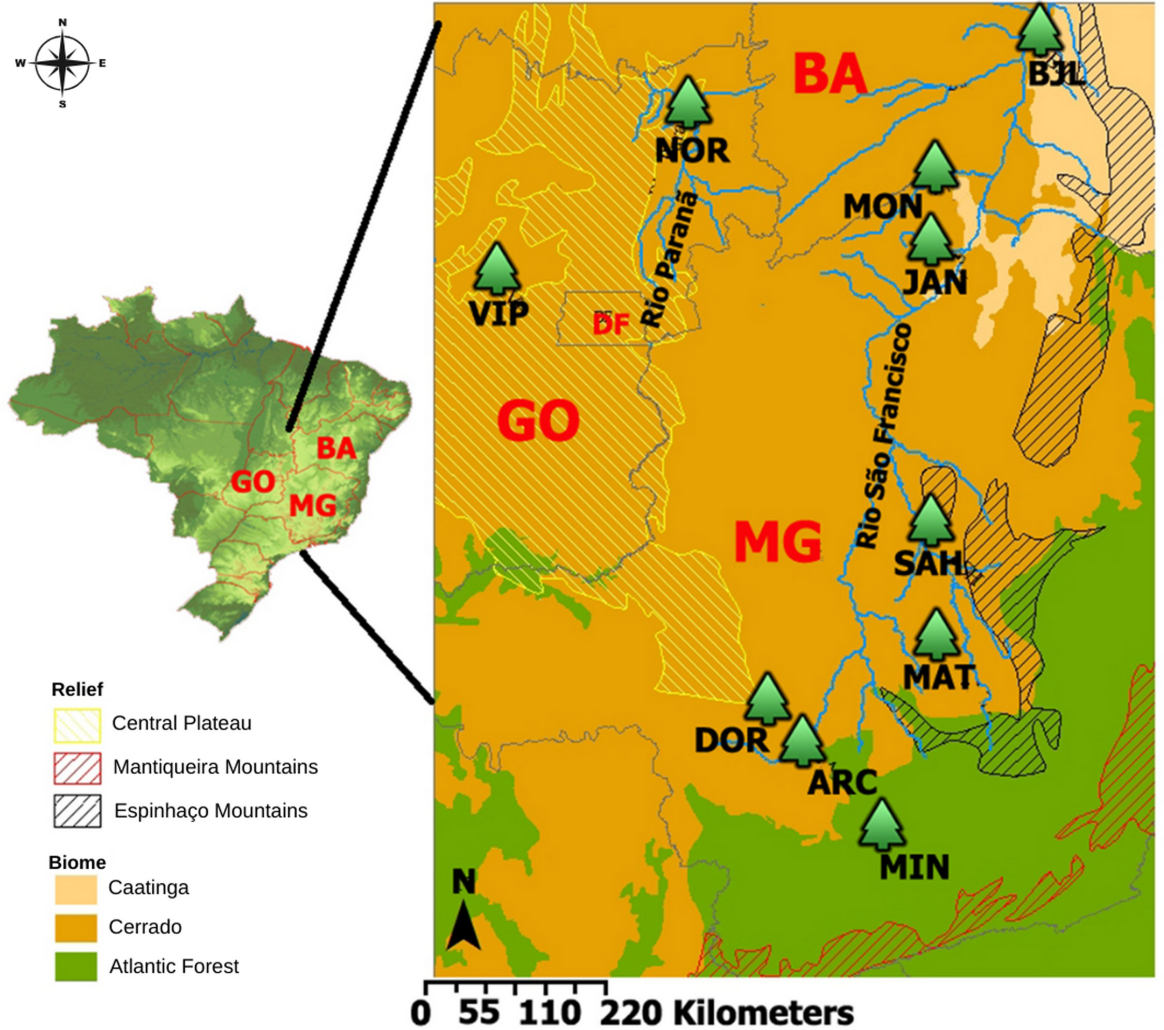
Location of sampled populations of *Ceiba pubiflora*. GO: Goiás State, MG: Minas Gerais State, BA: Bahia State, DF: Distrito Federal. Classification according to Veloso et al. [33] and Ab’Saber [34]. The Brazilian map’s relief is reprinted from Miranda [35] under a CC BY license, with permission from Embrapa, original copyright 2023.

Leaf samples of *Ceiba pubiflora* were collected from ten locations across the states of Minas Gerais, Bahia, and Goiás (Table 1 and Fig 1). All individuals of *Ceiba pubiflora* within the fragments were sampled, resulting in N (population size) being equivalent to n (sample size). The variation in sample size among populations was primarily due to differences in the population size of *Ceiba pubiflora* in their respective habitats. Accessing the limestone outcrops often required rock climbing (S1 Fig) and other demanding methods for sampling (e.g. use of a slingshot). Consequently, this habitat type has historically been underrepresented in population studies [11], leading to limited data on phytosociological surveys [2]. In some limestone outcrop areas, population densities occasionally drop to less than one individual per hectare. The collected leaf samples were carefully placed in plastic bags containing silica gel for tissue dehydration and transported to the laboratory.

**Table 1:** Locations, sample size (n) and domain of *Ceiba publiflora* populations. Classification of domains according to Veloso et al. [33] and Ab’Saber [34]. BA: Bahia State, MG: Minas Gerais State, GO: Goiás State, NOR: Nova Roma, ARC: Arcos, DOR: Doresópolis, BJL: Bom Jesus da Lapa, SAH: Santo Hipólito, MAT: Matozinhos, JAN: Januária, VIP: Vila Propício, MIN: Minduri, MON: Montalvânia.

Locations	Codes	Coordinates (S/W)		n	Domain
Bom Jesus da Lapa, BA	BJL	13°03’13.9”	43°17’28.6”	03	Caatinga
Montalvânia, MG	MON	14°28’21.0”	44°22’14.8”	16	Caatinga
Januária, MG	JAN	15°08’03.1”	44°14’58.4”	16	Caatinga
Nova Roma, GO	NOR	13°42’25.7”	46°51’14.5”	10	Cerrado
Vila Propício, GO	VIP	15°29’14.0”	48°51’52.7”	19	Cerrado
Santo Hipólito, MG	SAH	18°17’23.7”	44°11’13.9”	16	Cerrado
Matozinhos, MG	MAT	19°33’08.9”	44°04’14.4”	13	Atlantic Forest
Arcos, MG	ARC	20°19’52.5”	45°34’43.8”	03	Atlantic Forest
Doresópolis, MG	DOR	20°18’25.2”	45°55’08.5”	03	Atlantic Forest
Minduri, MG	MIN	21°39’53.4”	44°37’50.6”	05	Atlantic Forest

The sampled populations are forest fragments of Submontane and Montane Seasonal Deciduous Forests located on carbonate rock outcrops (S2 Fig). Only the MIN population (Minduri, state of Minas Gerais) is situated in a Baixomontana Semideciduous Seasonal Forest rather than on limestone outcrops. These populations are found within the Cerrado, Atlantic Forest and Caatinga domains [33, 34]. Their surroundings generally consist of Seasonal Deciduous Forests, surrounded by an anthropized matrix, featuring areas destined for monoculture cultivation or pasture. The access to genetic heritage of this research was registered in the National System of Management of Genetic Heritage and Associated Traditional Knowledge (*Sistema Nacional de Gestão do Patrimônio Genético e do Conhecimento Tradicional Associado*–SisGen) and identified by the code A77014D.

### DNA extraction and PCR-ISSR

The genomic DNA extraction from *Ceiba pubiflora* trees followed Doyle and Doyle’s method [36]. Polymerase Chain Reaction (PCR-ISSR) was employed to amplify DNA fragments in 12 μL, consisting of approximately 10 ng of each DNA sample, 1x reaction buffer (500 mM Tris-HCl pH 8.0, 200 mM KCl, 2.5 mg. mL^-1^ BSA, 200 mM Tartrazine and 1% Ficoll), 2.6 mM MgCl_2_, 0.25 mM of each dNTP, 0.125 U Taq DNA Polymerase (Phoneutria) and 0.4 μM ISSR primer. The PCR amplification program consisted of an initial denaturation at 94°C for 2 minutes, followed by 37 cycles with denaturation at 94°C for 15 seconds, annealing at 42°C for 30 seconds, and extension at 72°C for 1 minute. A final extension was performed at 72°C for 7 minutes. Eight ISSR primers (Table 2) showed a clear amplification profile of DNA fragments. Subsequently, the amplified DNA fragments were separated by electrophoresis in a 1.5% agarose gel in 0.5X TBE solution (90 mM Tris, 92 mM boric acid, and 2.5 mM EDTA), and stained with ethidium bromide. The gel was then photographed under light, and the UVP Doc-It-LS image analysis software was used for data analysis. A binary matrix was generated from gel genotyping, indicating the presence (1) or absence (0) of each fragment, which was utilized to calculate genetic diversity estimates for *Ceiba pubiflora* populations. Only unambiguous data obtained from DNA fragments were considered for the analysis.

**Table 2:** Primers used for amplification of genomic DNA from *Ceiba pubiflora*, along with their respective sequences, number of amplified loci, number of polymorphic loci (N° of PL), and Polymorphic Information Content (PIC) values. Where: R = purine (A or G) and Y = pyrimidine (C or T); B = (C, G, T i.e., not A); D = (A, G, T i.e., not C).

*Primer ISSR*	Sequence (5’– 3’)	N° of Loci	N° of PL	PIC
UBC 825 (AC)8T	ACA CAC ACA CAC ACA CT	14	14	0.337
UBC 835 (AG)8-YC	AGA GAG AGA GAG AGA GYC	8	7	0.439
UBC 842 (GA)8-YG	GAG AGA GAG AGA GAG AYG	11	10	0.414
UBC 857 (AC)8YG	ACA CAC ACA CAC ACA CYG	13	13	0.309
UBC 888 BDB(CA)7	BDB CAC ACA CAC ACA CA	19	19	0.356
OMAR (GAG)4-RC	GAG GAG GAG GAG RC	17	17	0.370
CHRIS (CA)7-YG	CAC ACA CAC ACA CAY G	7	7	0.346
UBC 841 (GA)8-YC	GAG AGA GAG AGA GAG AYC	6	6	0.379
TOTAL/Average		95	93	0.369

### Data analysis

#### Genetic diversity

The characterization of genetic variability was performed using the POPGENE v.1.32 program [37] with parameters suitable for analyzing dominant diploid data. We calculated the optimal number of loci following Kruskal [38] by assessing the correlation between the original and simulated genetic distance matrix (S3 Fig). Expected heterozygosity (He) was determined by the formula: *He = 1 - ∑p*_*i*_^*2*^, where *pi* is the frequency of band i. Additionally, the Shannon index (*I*) of phenotypic diversity was estimated using the formula: I = -∑*pi* Ln*pi/n*, where *pi* denotes the band frequency, and *n* is the number of evaluated markers.

Total heterozygosity (*H*_*T*_), average genetic diversity within populations (*H*_*S*_), and gene flow (*Nm*) were estimated using the indirect calculation provided by the formula: *Nm = 0*.*25 (1 –G*_*ST-B*_*)/G*_*ST- B*_ [39]. The population differentiation coefficient (G_ST-B_) required for this calculation was obtained via Bayesian analysis utilizing the HICKORY v.1.1 program [40]. Additionally, gene flow was estimated between population pairs using the same indirect calculation method, aiming to identify the populations contributing the most to the allele exchange between them, with the assistance of the HICKORY program. The percentage contribution of each population to the total gene flow (*Nm*) between population pairs was determined. Furthermore, the Mantel test was conducted using the PC-Ord 4.14 program [41] to verify a correlation between the gene flow data matrix among population pairs and the geographic distance matrix.

#### Genetic structure

The Analysis of Molecular Variance ‐ AMOVA was utilized to partition the total genetic variance into the covariance components attributed to differences within and between individuals, as well as between subpopulations within populations [42].

The Bayesian approach [43] was employed to obtain the θ^B^ genetic divergence index between pairs of populations, with the assistance of the HICKORY v1.1 program [40]. The values of total genetic heterozygosity (H_T-B_) and mean heterozygosity within the population (H_S-B_) was also obtained using the Bayesian method. The θ^B^ value was determined under four models for dominant markers: Free model (Free *f*), estimating inbreeding values; model θ^B^ = 0 and model *f* = 0, where θ^B^ and *f* were respectively fixed at zero; and Full model, encompassing all previous possibilities. Simulations were conducted using the Markov Monte Carlo chain (MCMC) [44]. The model selection was based on the observation of DIC (Deviance Information Criterion) values, adopting the one with the lowest DIC value [45]. The representation of genetic divergences (θ^B^) was simplified by constructing dendrograms using the UPGMA clustering method ‐ Unweighted Pair-Group Method, Arithmetic Average [46], and adopting the SAHN (Sequential Agglomerative, Hierarchical & Nested Clustering) routine. This analysis was performed using the NTSYS v2.11 program [47]. The cophenetic correlation (r_C_) was performed using the PAST 4.03 program [48].

The Mantel test was applied using the PC-Ord 4.14 program [41] to verify the presence of a correlation between the genetic distance matrix (θ^B^) and the geographic distance matrix of population pairs. Additionally, this test was utilized to examine the correlation between geographic distance classes and genetic distances (θ^B^) to explore the spatial structure pattern between populations through a multivariate correlogram. The geographic distance classes were defined to maintain the same number of connections between populations, following Telles et al. [49].

To infer the number of genetic groups (K) represented by the sampled populations, Bayesian analysis (Structure v.2.2) was used [50]. The number of genetic groups (K) ranged from k = 1 to k = 10, with twelve independent runs for each K. The unmixed ancestry model (*no admixture*) and the frequency of correlated alleles were applied. Each run consisted of 100,000 Monte Carlo Markov Chain (MCMC) repetitions, and a burn-in of 50,000. The number of K populations was obtained by calculating the average of each K by the *Log-likelihood*, *’log of probability*’ (LnP(D)) model, where the number of genetic groups is expected to be the maximum K value observed or the smallest negative number of Ln [50].

The Barrier v.2.2 program [51] was used to indicate genetic discontinuities in space employing the Delaunay triangulation method and the Monmonier algorithm [51]. Geographical distances obtained via the Global Positioning System (GPS) and genetic distances (θ^B^) were used for this analysis. Operational Units (OUs) for the genetic conservation of the *Ceiba pubiflora* populations were proposed based on the diversity and genetic structure data, along with the Mantel correlogram as proposed by Diniz-Filho and Telles [16]. These authors suggest the definition of Operational Units based on the spatial pattern of genetic variability, obeying a minimum distance between populations to ensure they can be considered genetically independent.

## Results

### ISSR markers

The eight ISSR primers utilized in this study successfully generated 95 loci, a sufficient number of loci for estimating the genetic diversity of *Ceiba pubiflora* (S3 Fig). Each locus displays a distinct profile for identification in all individuals, of which 93 loci were polymorphic (97.9%). The number of loci ranged from 6 to 19, with an average of 11 loci (Table 2), and their sizes ranged from 300 to 2,500 bp. The Polymorphic Information Content (PIC) value, a measure of genetic diversity, ranged from 0.309 to 0.439, with an average of 0.369. In 72 loci, the PIC value exceeded 0.300, indicating that these markers are highly efficient in revealing polymorphisms between individuals.

### Genetic diversity

The total genetic heterozygosity (H_T_) of all populations, assuming Hardy-Weinberg equilibrium, was 0.345 (±0.017), while the mean within-population heterozygosity (H_S_) was 0.253 (±0.007). The coefficient of population differentiation (G_ST_) [52] was 0.267, indicating moderate genetic differentiation among populations, and the historical gene flow (Nm) between populations was 1.37 migrants per generation. The genetic diversity [53] and the Shannon index for the species were 0.364 and 0.541, respectively. The populations Santo Hipólito (SAH), Januária (JAN), and Montalvânia (MON) exhibited the highest levels of genetic diversity (Table 3 and S4 Fig). The percentages of polymorphic loci in these populations were 90.53%, 86.32%, and 84.21%, respectively. On the other hand, the BJL, MIN, ARC, and NOR populations showed lower levels of genetic diversity compared to the other populations Bom Jesus da Lapa (BJL) and Mindurí (MIN) populations standing out for their particularly low values of genetic diversity, with He values of 0.101 and 0.143, and percentages of polymorphic loci at 24.21% and 26.32%, respectively. Notably, these locations are among those with the smallest population size (Table 1).

**Table 3:** Estimates of genetic diversity in ten populations of *Ceiba pubiflora*. He: genetic diversity of Nei [52]; *I*: Shannon index; %PL: percentage of polymorphic loci; (): standard deviation. For the MON population, 11 individuals were genotyped.

Population	He	*I*	N° Polymorphic Loci	% PL
BJL	0.101 (0.178)	0.146 (0.260)	23	24.21
MON	0.325 (0.177)	0.478 (0.243)	80	84.21
JAN	0.338 (0.166)	0.496 (0.227)	82	86.32
NOR	0.235 (0.202)	0.349 (0.288)	60	63.16
VIP	0.271 (0.184)	0.406 (0.257)	74	77.89
SAH	0.348 (0.155)	0.512 (0.207)	86	90.53
MAT	0.298 (0.182)	0.444 (0.247)	80	84.21
ARC	0.246 (0.217)	0.356 (0.307)	56	58.95
DOR	0.257 (0.214)	0.373 (0.303)	59	62.11
MIN	0.143 (0.198)	0.210 (0.289)	25	26.32
Total	0.256 (0.187)	0.377 (0.263)	94	97.89

**Fig 2:**
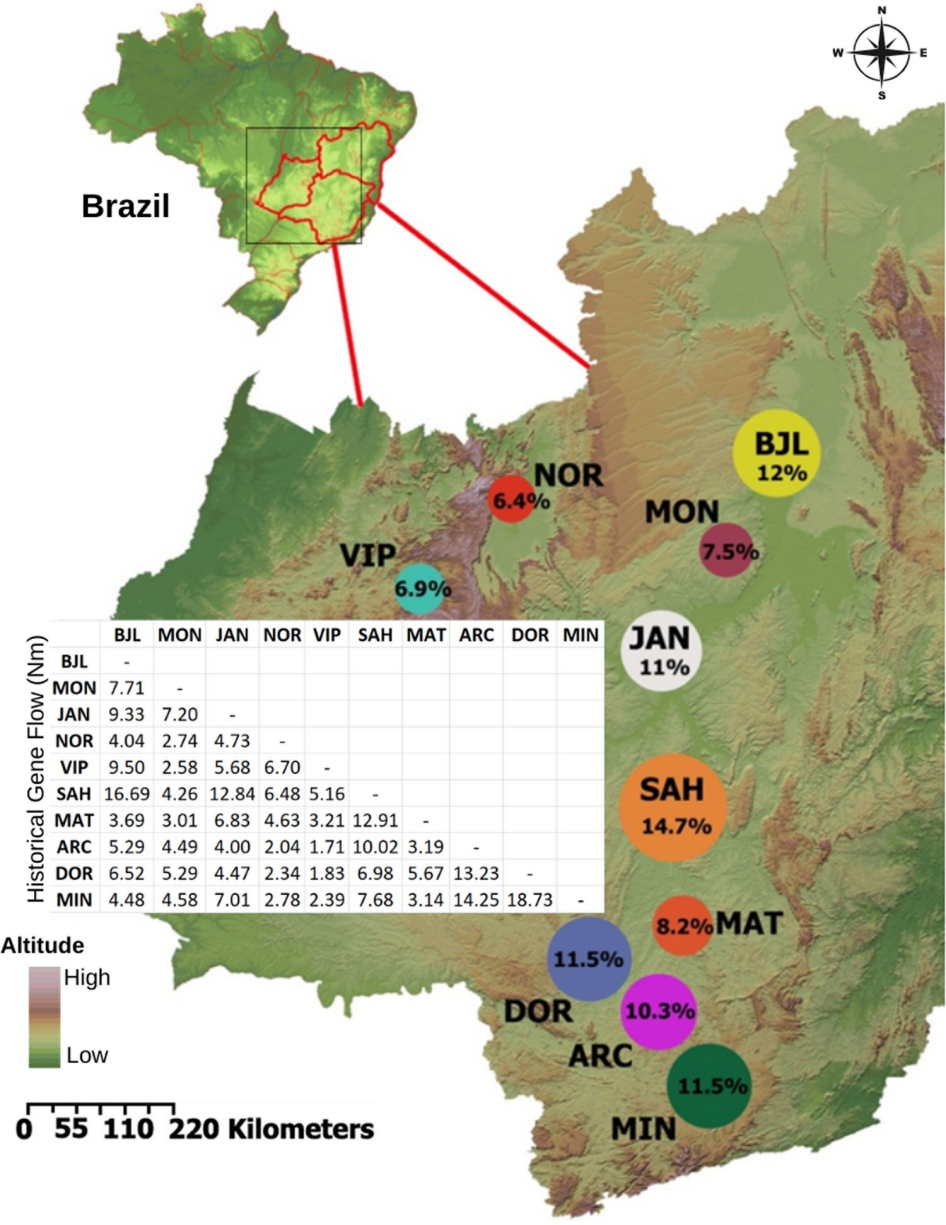
Distribution of historical gene flow contribution between populations of *Ceiba pubiflora*. The circles are proportional to the Nm contribution (%). The matrix of gene flow values (Nm) between all pairs of populations is provided on the side. The Brazilian map’s relief is reprinted from Miranda [35] under a CC BY license, with permission from Embrapa, original copyright 2023.

A higher historical gene flow (Nm) was observed among the SAH, BJL, DOR, MIN, and JAN populations (Fig 2 and S1 Table). The SAH and BJL populations stand out because they participated with more than 1/4 of the gene flow among all populations. On the other hand, the NOR and VIP populations exhibited the lowest values of gene flow with the other populations. However, the Mantel test showed no significant correlation between the matrices of gene flow (Nm) and geographic distance among population pairs (r = –0.08; p = 0.57).

### Genetic structure

The analysis of molecular variance (AMOVA) indicates a more significant proportion of genetic variability between individuals within populations (91.51%) than between populations (Table 4). AMOVA revealed a low level of genetic differentiation among the ten populations of *Ceiba pubiflora* with an *F*_ST_ value of 0.084 (p < 0.0001).

**Table 4:** Molecular analysis of variance (AMOVA) in populations of *Ceiba pubiflora*. GL: degrees of freedom; SQ: sum of squared deviations; *F*_ST_: genetic differentiation.

Source of variation	GL	SQ	Variance components	Total Variation (%)	*P*
Between populations	9	207.247	1.227	8.49	< 0.0001
Within populations	87	978.693	13.225	91.51	< 0.0001
Total	96	1185.940	14.452	100	
*F*_ST_	0.084				

The Full model was employed in the Bayesian approach as it demonstrated the lowest DIC value (2832) (Table 5). The total genetic heterozygosity (H_T-B_ = 0.391) and mean heterozygosity within the population (H_S-B_ = 0.357), calculated by the Bayesian method, closely resembled those obtained through Nei’s calculation (0.345 and 0.253, respectively). However, the coefficient of population differentiation was lower (G_ST-B_ = 0.087) than that found using Nei’s estimate (0.267).

**Table 5:** Estimation models for the genetic distance θ^B^, tested by Bayesian analysis among populations of *Ceiba pubiflora*. SD: standard deviation; CI: confidence interval; DIC: deviance information criterion.

Model	θ^B^			*F*			DIC
	Average	SD	CI (97.5%)	Average	SD	CI (97.5%)	
Full	0.09	0.01	(0.07; 0.12)	0.71	0.23	(0.15; 0.99)	2832
*f* = 0	0.06	0.01	(0.05; 0.08)	–	–	–	2874
θ^B^ = 0	–	–	–	0.75	0.12	(0.49; 0.96)	3229
Free *f*	0.12	0.01	(0.09; 0.15)	0.50	0.28	(0.02; 0.97)	2962

From the Full model, the genetic distances θ^B^ between pairs of populations were estimated by Bayesian analysis (Table 6). The MIN and DOR populations exhibited the lowest genetic distance value between them (0.026), while the ARC and VIP populations had the highest value (0.241). The average θ^B^ value of all combinations was 0.100, with an average geographic distance of approximately 490 km. The Mantel test revealed the existence of a positive and significant correlation (r = 0.425; p = 0.008) between genetic and geographic distances among populations (S5 Fig).

**Table 6:** Genetic distance θ_B_ (below the diagonal) and geographic distance (Km) between population pairs of *Ceiba pubiflora* (above the diagonal).

	NOR	ARC	DOR	BJL	SAH	MAT	JAN	VIP	MIN	MON
NOR	0	738.25	732.12	376.87	578.59	712.83	333.97	288.66	908.39	280.63
ARC	0.194	0	81.0	811.07	262.62	175.00	558.38	641.84	178.51	662.71
DOR	0.172	0.037	0	822.07	283.64	208.46	556.93	620.70	202.43	669.92
BJL	0.112	0.083	0.069	0	561.92	701.74	266.06	638.89	938.78	165.5
SAH	0.075	0.049	0.068	0.030	0	142.52	309.29	588.49	375.82	428.05
MAT	0.100	0.139	0.083	0.122	0.038	0	451.66	686.32	238.64	570.39
JAN	0.102	0.114	0.104	0.053	0.040	0.072	0	485.82	680.18	121.14
VIP	0.072	0.241	0.221	0.051	0.096	0.138	0.089	0	817.72	497.92
MIN	0.149	0.034	0.026	0.092	0.062	0.140	0.068	0.178	0	801.38
MON	0.160	0.101	0.086	0.063	0.106	0.145	0.068	0.172	0.100	0

**Fig 3:**
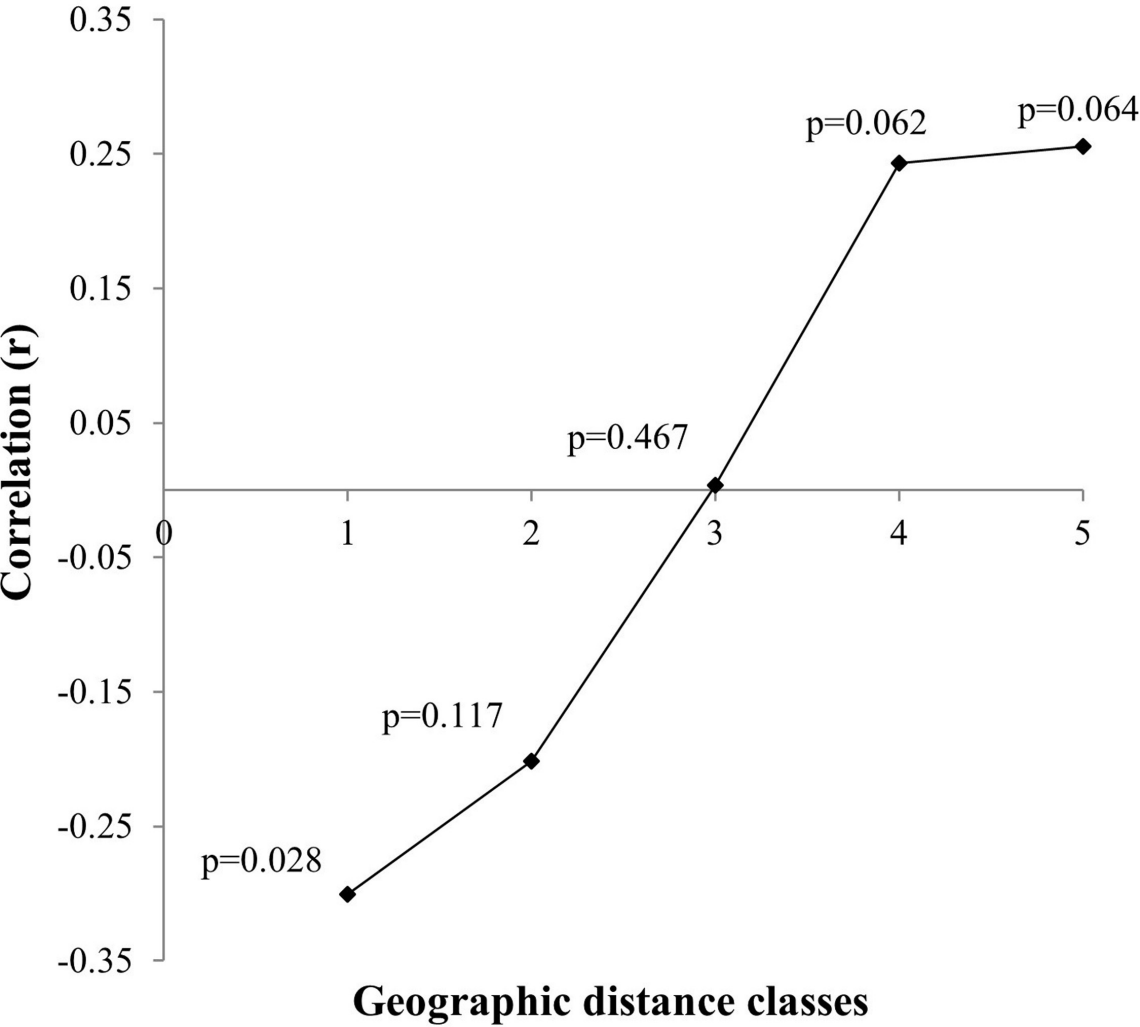
Correlation between genetic distance θ^B^ and five geographic distance classes (class 1: 0–250 km; class 2: 251–400 km; class 3: 401–580 km; class 4: 581–710; class 5: 711 -) for *Ceiba pubiflora*.

The multivariate correlogram (Fig 3) shows a negative and significant correlation in the first geographic distance class (r = –0.3 00; p = 0.028). For the other distance classes, the values were higher but not statistically significant (p > 0.05). The interception of the X axis occurred in the third class of distance, between 401 and 580 km. This intercept indicates the geographic distance where populations can be considered as genetically independent groups, suggesting their designation as Operating Units (OU).

**Fig 4:**
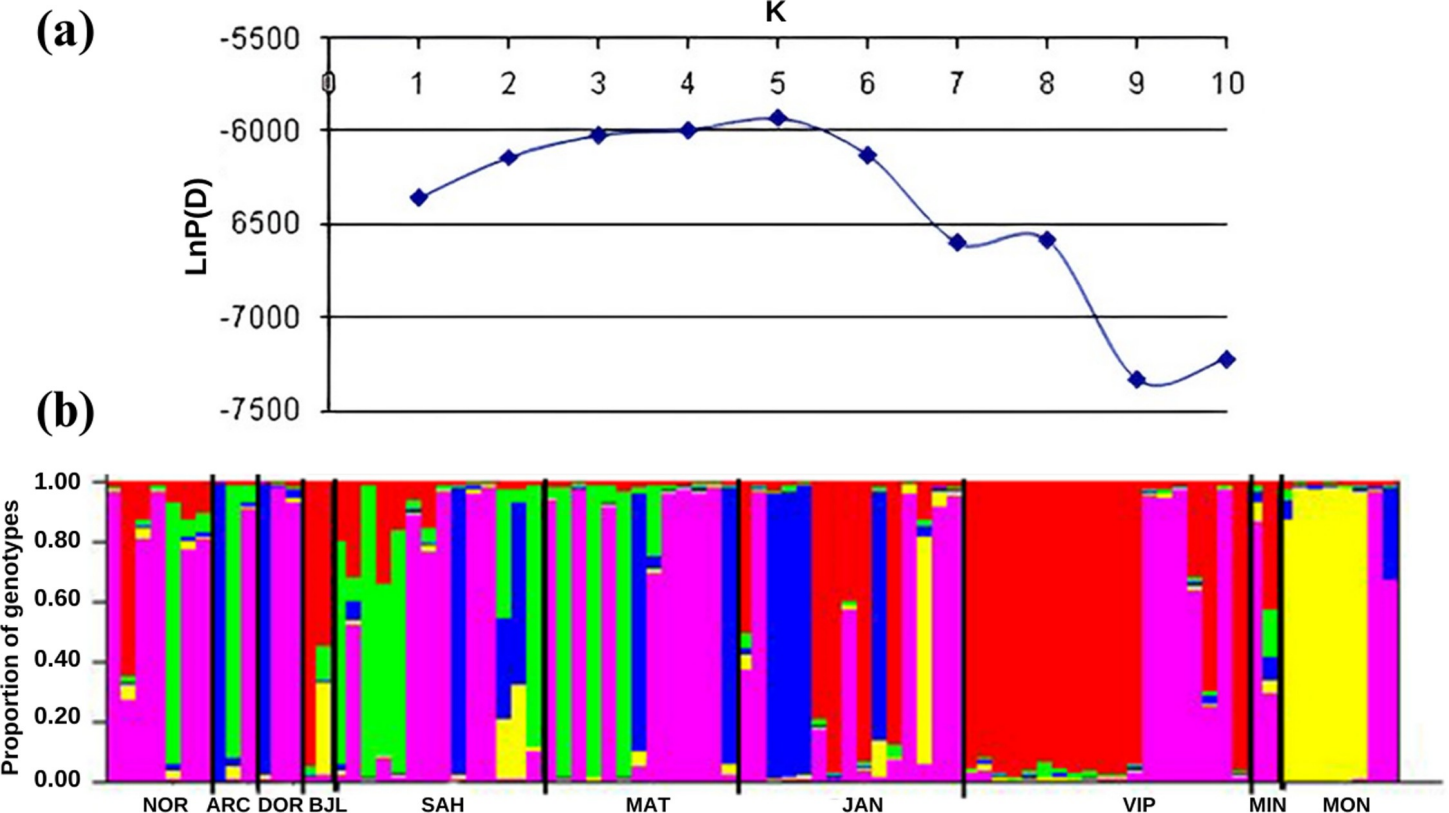
Values of genetic clusters (K) and mean probability log (LnP(D)), obtained by Bayesian analysis (A), and the proportion of genotypes in the sampled populations delimited by the dark vertical bar (B).

The Bayesian analysis revealed the presence of five distinct genetic groups (Fig 4A and 4B).

The value of K reveals the real number of genetic groups. In this study, the highest observed value of LnP(D) indicates a grouping structure of the ten populations into five genetic clusters (Fig 4A), represented by the colors in the graph (Fig 4B).

**Fig 5:**
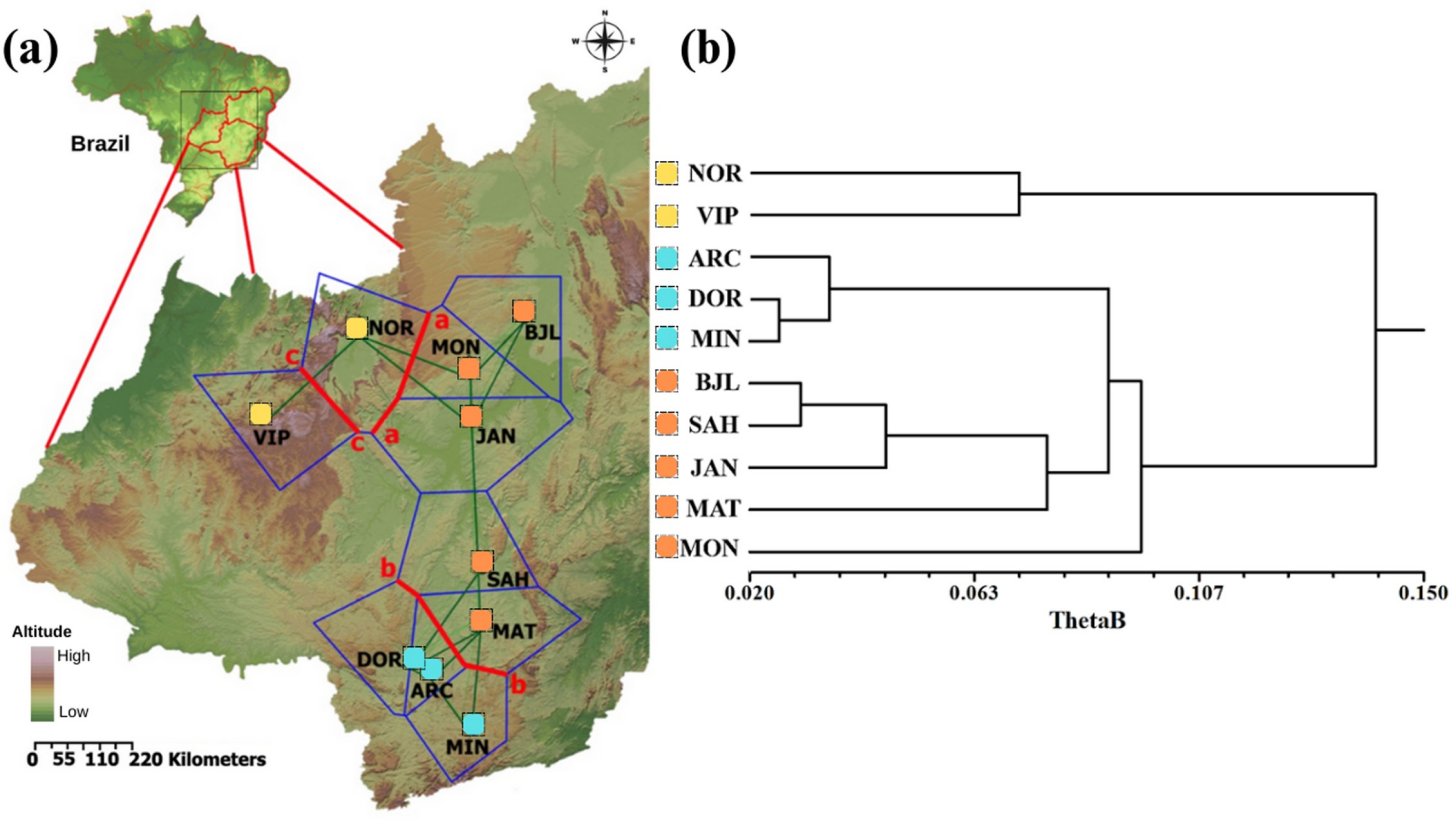
Relief map of the sampled populations of *Ceiba pubiflora* and the Delaunay triangulation network (blue lines) (A) and UPGMA dendrogram based on genetic distance (θ^B^) between populations (B). The red lines (a, b, and c) in (A) indicate the genetic discontinuities between population sets. The cophenetic correlation was r_C_ = 0.805. The Brazilian map’s relief is reprinted from Miranda [35] under a CC BY license, with permission from Embrapa, original copyright 2023.

Delaunay triangulation indicated the existence of at least three barriers (Fig 5A). The most prominent barrier (1) separates the populations of Minas Gerais State and Bahia from the populations of Goiás (Fig 5A, barrier ‘a’). The second barrier (2) isolates the populations of the south of Minas Gerais from the other populations (Fig 5A, barrier ‘b’). The third barrier (3) separates the VIP population from the other populations (Fig 5A, barrier ‘c’), based on the values of θ^B^.

In addition to the geographical distance, which naturally separates the sampled populations, other possible barriers to gene flow between populations can be identified. These would be: (a) Central Plateau or Paranã River valley and extensive areas of cultivation; (b) near the Espinhaço chain or São Francisco River valley and, change in vegetation physiognomy, ecotonal region of Semideciduous Forest formations of the Atlantic Forest domain (Fig 1); (c) extensive areas with anthropic intervention and the Central Plateau. The dendrogram also showed that geographically close populations present lower genetic distances (Fig 5B).

The dendrogram shows the formation of two main genetic groups. One group includes the populations located in the state of Goiás (NOR and VIP), while the other group includes the remaining populations. Within this second group, there is further subdivision into two subgroups. One subgroup is composed of populations under the influence of the Atlantic Forest domain (ARC, DOR and MIN), which are geographically close to each other. The other subgroup is formed by populations located to the north (BJL, SAH, JAN, and MAT), which are genetically similar to the subgroup located to the south, including the Montalvânia population (MON). However, the MON population shows some genetic distance from both subgroups, indicating a degree of genetic differentiation. These results are consistent with the findings of the Mantel test, which revealed a correlation between genetic and geographic distances.

## Discussion

Our study using ISSR molecular markers for conservation proposes showed high genetic diversity within populations of *Ceiba pubiflora*. The findings support the hypothesis that the remaining populations of this species retain genetic variability, which is vital for their long-term survival. The genetic diversity indices, estimated from allele frequencies, provide valuable support for effective conservation measures. The use of ISSR markers in this study demonstrated their efficiency in quantifying the genetic diversity of *Ceiba pubiflora* and identifying polymorphism between individuals in populations, as evidenced by the results of the PIC (average of 0.369) and the calculation of the optimal number of loci (S3 Fig). Although the number of loci used in studies of genetic diversity of tree species with ISSR molecular marker is variable [31, 54], the calculation of the optimal number of loci and the PIC value have been used as a sampling reference [31].

*Ceiba pubiflora* exhibits high levels of genetic diversity for most of the analyzed populations, with the exception of the BJL and MIN populations. The average values of genetic diversity found for the species (He = 0.364; G_ST_ = 0.267) are close to the estimated values for species with crossbreeding system (H_POP_ = 0.27; G_ST_ = 0.22) and anemochoric dispersion (H_POP_ = 0.27) [54]. The percentage of polymorphic loci is a parameter commonly used in genetic conservation studies of populations [31, 55]. In this study, it was evident that half of the studied populations exhibited a percentage of polymorphic loci below 70% (NOR, ARC, DOR, BJL, and MIN), and the populations of BJL and MIN stood out with shallow values of genetic diversity and polymorphic loci (He = 0.101 and 0.143; %PL = 24.21 and 26.32, respectively). These reduced values might be attributed to the small population size of these areas (N = 3 and 5, respectively). Indeed, populations with small demographic sizes that remain isolated for many generations can be subject to two critical genetic consequences: high levels of genetic drift and inbreeding. These factors effectively contribute to low genetic diversity within these populations and can lead to the loss of low-frequency alleles due to genetic drift [56].

In seasonal deciduous forests, the occurrence of the *Ceiba pubiflora* species is associated with limestone outcrops [26]. These outcrops often impose limits on the establishment of the species, resulting in a low population density. In Semideciduous Seasonal Forests (e.g., MIN), *Ceiba pubiflora* is rarely found, leading to a low population density (< 1 ind.ha^-1^), with individuals separated by greater distances compared to those occurring in limestone outcrops. Therefore, the combination of low population density and isolation may be critical factors contributing to the low levels of diversity in these populations. Similar genetic diversity data were found for threatened species with low population density, such as *Dimorphandra wilsonii* (P_LP_ = 40%; I = 0.190; He = 0.124) [29], and *Heptacodium miconioides*, a threatened species endemic to China (P_LP_ = 78.3%; I = 0.3760; He = 0.246) [57]. Another important factor that must be considered is the high rate of forest fragmentation, especially in the vicinity of the studied sites. Over the last two centuries, the central region of Brazil has experienced significant urban and agricultural expansion. Large cultivated areas, especially in the south of the States of Minas Gerais and Goiás, have intensified the isolation of populations on limestone outcrops and, consequently, impacted populations of *Ceiba pubiflora* occuring in semideciduous forests.

Forest fragmentation and habitat loss can lead to genetic variability erosion [58, 59]. This occurs due to changes in plant mating systems, causing inbreeding and reduced gene flow rates, leading to increased population differentiation. In disturbed environments, there may be a loss of genetic variability and fitness of the species, increasing the risk of local population extinction in the short term and, in the long term, limiting the evolutionary potential of the species in the face of climate and environmental changes [58, 60, 61]. However, long-lived tree species can have different responses depending on interactions with pollen and seed dispersal agents and the species’ colonization strategy [58]. Some trees may be more resistant to the effects of fragmentation than previously supposed [58, 62]. The limits of forest fragments often do not effectively represent the limits for cross-fertilization of tree species populations [63] since tropical trees generally evolved in a context of few individuals per area, separated by large distances between them, where the pollination system necessarily circumvents this factor. On the other hand, species of rare occurrence, such as *Ceiba pubiflora*, may be more susceptible to forest fragmentation, mainly due to the loss of individuals and habitat loss and impacts on their pollinating agents. Populations with low population density would be more threatened by their demographic status than by the low genetic diversity within their population [29].

Population size (N) may also explain the high levels of genetic diversity observed in the SAH, JAN and MON populations. In these populations, there is more density of individuals on the outcrop, and they are situated in better-conserved areas, with the JAN population even located within an ecological reserve (Parque Estadual Veredas do Peruaçu). However, most of these populations are under significant anthropic pressure, and their surrounding environments are gradually being degraded, mainly due to illegal timber harvesting for charcoal production and the expansion of livestock areas. Additionally, there is the potential for limestone extraction within these remnants, driven by the increasing demand required by urban expansion. Impacts that disrupt the species’ habitats and alter the behavior of *Ceiba pubiflora* pollinators can lead to a decline in genetic diversity within populations, as previously discussed.

Despite the geographic distances that separate some populations (with an average of 490 km), historically gene flow has persisted between them, with an average of two migrants per generation (Nm = 2.62). *Ceiba pubiflora* is primarily pollinated by hummingbirds and, to a lesser extent, by butterflies and bats [26], while its seeds are dispersed by the wind. The abundant production of seeds, which are lightweight and hairy that facilitate wind dispersal, along with the hummingbird’s capacity to traverse the distances that separate trees during foraging, enables extensive alleles recombination within and between population. As a result, the observed levels of gene flow and the low differentiation between populations are indicative of historical and substantial allele recombination, essential for the allele homogenization across populations [64].

The gene flow dynamics among the studied populations showed a pronounced flow in the southern region sampled and within the São Francisco River valley (see Fig 2). The southern area (DOR, ARC, MIN) and the SAH and BJL populations may serve as significant sources of gene flow. In regions where the MAT and DOR populations are situated, limestone outcrops likely serve as pathways for historical gene flow. These colonization pathways can also explain the flow observed between distant populations (e.g. NOR, SAH, MIN). Therefore, each limestone outcrop has connected populations over generations through gene flow.

The MIN population, situated within the Semideciduous Seasonal Forest and not on limestone outcrops, likely experiences distinct environmental selection pressures compared to outcrops populations. This scenario potentially leads to the accumulation of different and adapted genotypes for genetic restoration purposes [65]. In an overview, populations of *Ceiba pubiflora* that are closer to each other exhibited a higher propensity for allele exchange, probably influenced by wind-mediated seed dispersal. Although the VIP, NOR, and MON populations made relative contributions to gene flow dynamics between populations, they hold significance in upholding genetic diversity *in situ*.

The gene flow dynamics between populations suggest that the São Francisco River Basin could have been a conceivable migration route for *Ceiba pubiflora*. This result indicates that this region might have functioned as a refuge for the species during the last glaciation period (18.000–12.000 years ago) and that subsequent colonization efforts radiated from this location. It is hypothesized that the Seasonal Deciduous Forests (SDFs) represent remnants that expanded during the arid and cold Pleistocene period, while the more humid forests retracted to less arid zones. As humidity levels increased, the SDFs retracted, yielding humid forests, whereas forests on limestone outcrops preserved favourable edaphic conditions for the SDFs [7, 8, 66, 67].

The results showed low genetic differentiation among populations, confirmed by molecular analysis of variance (*F*_ST_ of 0.084) and the Bayesian (θ^B^ of 0.096). This pattern aligns with the typical trend observed in perennial, cross-fertilization species, which tend to exhibit higher diversity within populations [54, 68]. Furthermore, species that inhabit limestone outcrops develop under specific environmental conditions, fostering a selection of genotypes adapted to these unique conditions. This, in turn, would also explain the observed low population differentiation since populations subjected to similar environmental conditions tend to harbor similar genotypic compositions [69].

The SAH, MAT, JAN, VIP, and MON populations showed a lower genetic structure than others. These populations contain more frequent but not exclusive alleles, indicating an ancient connection between populations. Moreover, the multivariate correlogram indicated that beyond the 400 km threshold, populations below this distance demonstrate significant genetic similarity, effectively forming the same Operational Unit. This distance refers to establishing sampling strategies for conserving and managing genetic diversity in *Ceiba pubiflora* [16, 17, 70]. To optimally capture the genetic diversity within *Ceiba pubiflora* populations, identifying of Operational Units for management or seed collection areas should consider a population separation of 400 km. For example, the SAH, MON, and VIP populations, along with the population located in the southern of Minas Gerais (e.g., MIN), can be identified as Operational Units for *Ceiba pubiflora*, as proposed by Diniz-Filho and Telles [16].

Potential barriers to gene flow were identified for *Ceiba pubiflora*, encompassing the depression of the São Francisco River basin, the Central Plateau, and the Paranã valley. Across the study regions, in addition to the natural barriers and the geographic distance separating the forest remnants, extensive cultivated areas and urban agglomerations are observed. These factors, added to the damage caused by forest exploitation and limestone extraction, put the natural populations of *Ceiba pubiflora* and the ecological interactions within these peculiar environments at risk. Therefore, these recognized barriers and genetic discontinuities between populations should be considered to establish priority areas for *in situ* conservation and seed collection to establish germplasm banks [12]. Finally, we recognize the value of a more comprehensive sample size across populations, particularly for future phylogeographic studies of the species. Also, it is important to emphasize that our study contributes valuable insights into the genetic diversity of *Ceiba pubiflora* within its predominant occurrence areas.

### Conservation strategies

Limestone outcrops constitute areas that are under constant anthropic pressure. The extraction of limestone for cement production contributes to the degradation of a distinctive habitat which supports ecologically important species. Given their high genetic diversity levels, areas such as MAT, SAH, and MON should be a priority for conservation. This is imperative because they are within zones experiencing pronounced deforestation and limestone extraction. Populations within ecotonal areas (e.g., ARC, MIN, NOR) also warrant conservation. These populations harbor individuals with adaptive traits for such habitats [71] and have a historical record of human-induced exploitation.

Aiming for the genetic conservation of *Ceiba pubiflora*, establishing specific Operational Units (OUs) dedicated to preservation becomes imperative. In the southern region of Minas Gerais State, an OU should be designated to safeguard individuals adapted to the transition towards more humid forests (e.g., MIN). Similarly, an OU within the northwestern region of Goiás State, coinciding with the transition from the Central Plateau (e.g., VIP), is essential. Another OU located in the central region of Minas Gerais State, encompassing populations with high genetic diversity and intense gene flow (e.g., SAH), would be prudent. A designated OU in the northernmost region of Minas Gerais and southern Bahia (e.g., MON) is warranted. This ecotonal expanse, linked to the Arboreal Caatinga, represents a habitat at risk of degradation and holds importance as an extension of the Atlantic Forest [67, 72].

## Supporting information

S1 Fig Rock climbing on a limestone karst outcrop (a) and the view from the top (b), showing the surrounding area. These images, provided by the authors, can be published under the Creative Commons Attribution License (CC BY 4.0).
(DOCX)

S2 Fig Limestone karst outcrop (a), the surrounded area within an anthropized matrix, destined for monoculture cultivation or pasture (b), and the Ceiba pubiflora tree (c and d). These images, provided by the authors, can be published under the Creative Commons Attribution License (CC BY 4.0)
(DOCX)

S3 Fig Pearson (r) correlation values as a function of the number of ISSR loci used to estimate the genetic diversity of *Ceiba pubiflora*. (DOCX)

S4 Fig Depiction of the genetic diversity (Nei’s He, values in parentheses) in populations of *Ceiba pubiflora*. Symbol size corresponds to He values. The Brazilian map’s relief is reprinted from Miranda (2005) under a CC BY license, with permission from Embrapa, original copyright 2023.
(DOCX)

S5 Fig Relationship between geographic distances (Km) and genetic distance (Theta B) for *Ceiba pubiflora* populations. (DOCX)

S1 Table Historical gene flow contribution (%) among populations of *Ceiba pubiflora*. The table presents the matrix of gene flow values (Nm) between all pairs of populations.
(DOCX)
