# Supplementary material for: Unraveling the genetic diversity of Ceiba pubiflora (Malvaceae) in isolated limestone outcrops: Conservation strategies
Source: PLoS One. 2024 Apr 1;19(4):e0299361. doi: 10.1371/journal.pone.0299361 (PMC10984428; doi:10.1371/journal.pone.0299361)
Supplement: S1 Fig — Rock climbing on a limestone karst outcrop (a) and the view from the top (b), showing the surrounding area. These images, provided by the authors, can be published under the Creative Commons Attribution License (CC BY 4.0). (DOCX) [file pone.0299361.s001.docx]

**Unraveling the genetic diversity of *Ceiba pubiflora* (Malvaceae) in isolated limestone outcrops: conservation strategies**


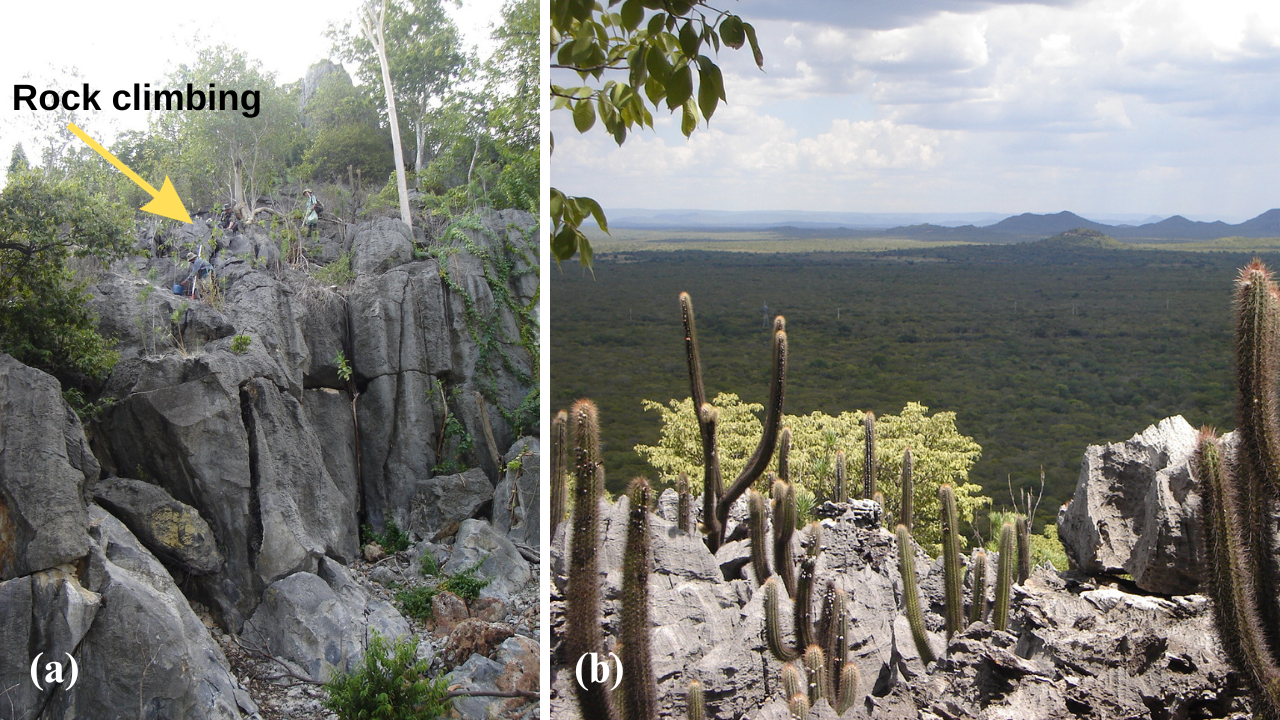


**S1 Fig. Rock climbing on a limestone karst outcrop (a) and the view from the top (b), showing the surrounding area.** These images, provided by the authors, can be published under the Creative Commons Attribution License (CC BY 4.0).
