# Supplementary material for: Unraveling the genetic diversity of Ceiba pubiflora (Malvaceae) in isolated limestone outcrops: Conservation strategies
Source: PLoS One. 2024 Apr 1;19(4):e0299361. doi: 10.1371/journal.pone.0299361 (PMC10984428; doi:10.1371/journal.pone.0299361)
Supplement: S2 Fig — Limestone karst outcrop (a), the surrounded area within an anthropized matrix, destined for monoculture cultivation or pasture (b), and the Ceiba pubiflora tree (c and d). These images, provided by the authors, can be published under the Creative Commons Attribution License (CC BY 4.0) (DOCX) [file pone.0299361.s002.docx]

**Unraveling the genetic diversity of *Ceiba pubiflora* (Malvaceae) in isolated limestone outcrops: conservation strategies**


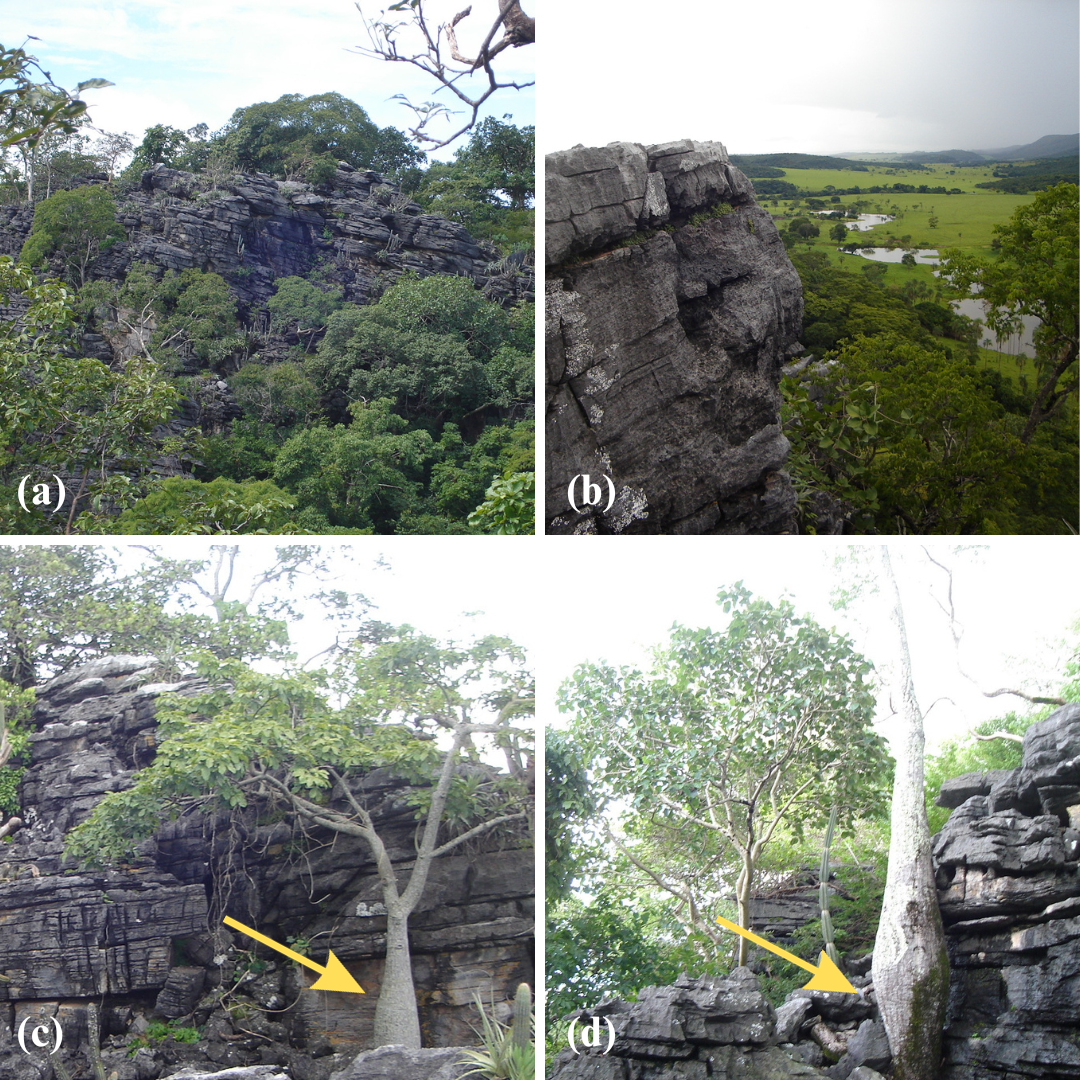


**S2 Fig. Limestone karst outcrop (a), the surrounded area within an anthropized matrix, destined for monoculture cultivation or pasture (b), and the *Ceiba pubiflora* tree (c and d).** These images, provided by the authors, can be published under the Creative Commons Attribution License (CC BY 4.0).
