# Supplementary material for: Unraveling the genetic diversity of Ceiba pubiflora (Malvaceae) in isolated limestone outcrops: Conservation strategies
Source: PLoS One. 2024 Apr 1;19(4):e0299361. doi: 10.1371/journal.pone.0299361 (PMC10984428; doi:10.1371/journal.pone.0299361)
Supplement: S3 Fig — (DOCX) [file pone.0299361.s003.docx]

**Unraveling the genetic diversity of *Ceiba pubiflora* (Malvaceae) in isolated limestone outcrops: conservation strategies**


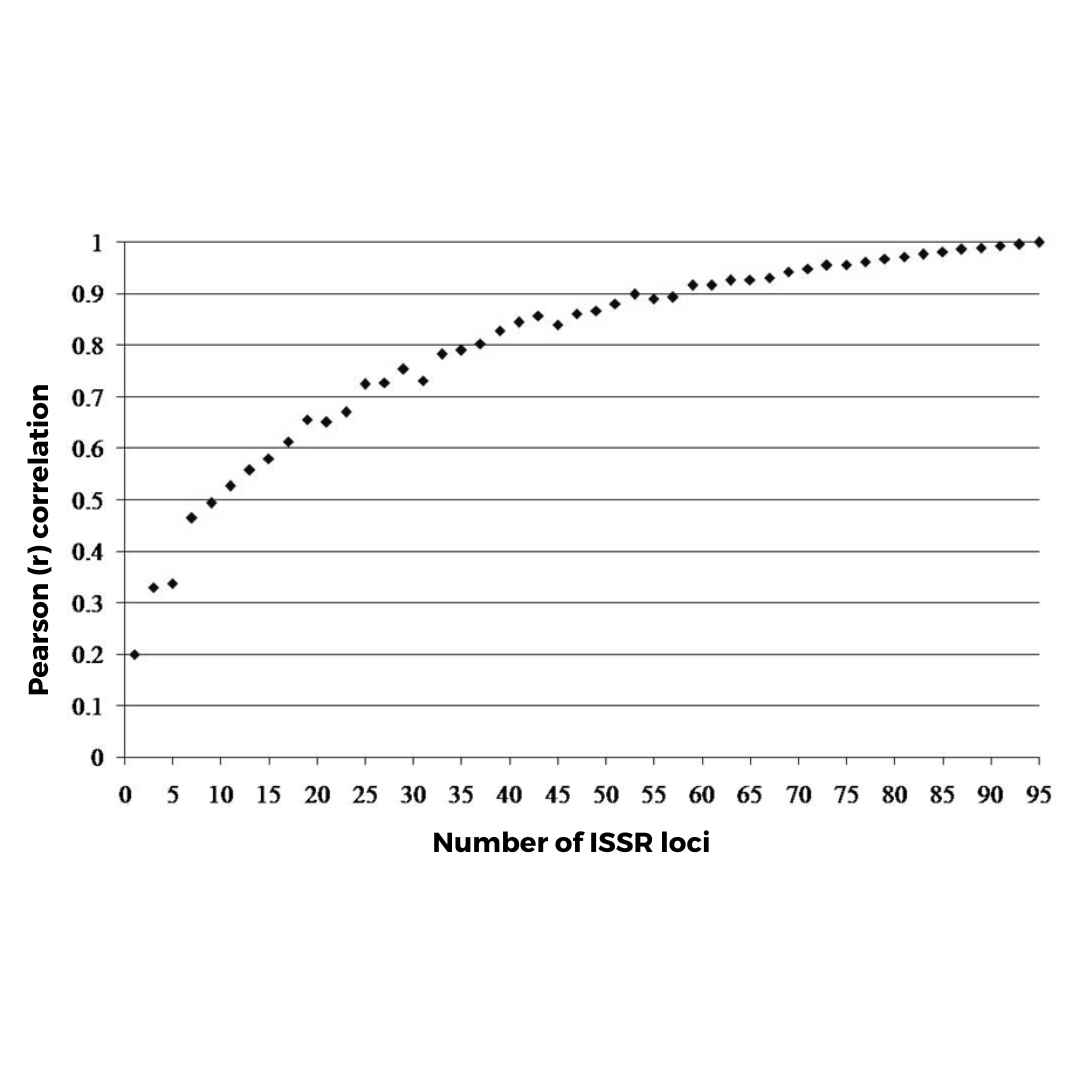


**S3 Fig. Pearson (r) correlation values as a function of the number of ISSR loci used to estimate the genetic diversity of *Ceiba pubiflora*.** Pearson (r) values close to 1.0 suggest a strong correlation between the original genetic distance matrix and the simulated genetic distance matrix, affirming that the number of loci obtained in this study (95 loci) was adequate for estimating the genetic diversity of *Ceiba pubiflora*.
