# Supplementary material for: Unraveling the genetic diversity of Ceiba pubiflora (Malvaceae) in isolated limestone outcrops: Conservation strategies
Source: PLoS One. 2024 Apr 1;19(4):e0299361. doi: 10.1371/journal.pone.0299361 (PMC10984428; doi:10.1371/journal.pone.0299361)
Supplement: S4 Fig — Symbol size corresponds to He values. The Brazilian map’s relief is reprinted from Miranda (2005) under a CC BY license, with permission from Embrapa, original copyright 2023. (DOCX) [file pone.0299361.s004.docx]

**Unraveling the genetic diversity of *Ceiba pubiflora* (Malvaceae) in isolated limestone outcrops: conservation strategies**


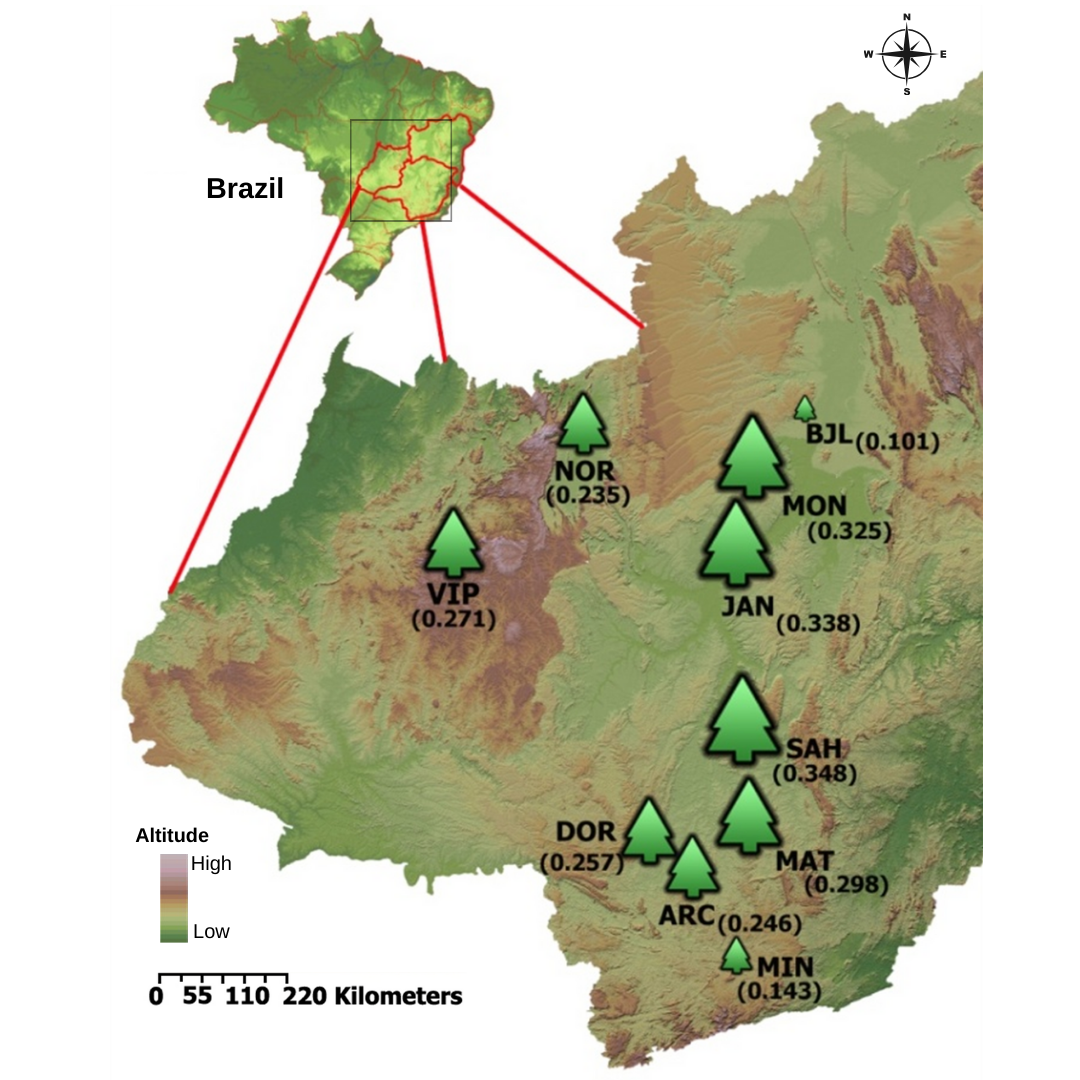


**S4 Fig. Depiction of the genetic diversity (Nei’s He, values in parentheses) in populations of *Ceiba pubiflora*.** Symbol size corresponds to He values. The Brazilian map’s relief is reprinted from Miranda (2005) under a CC BY license, with permission from Embrapa, original copyright 2023.
