# Supplementary material for: Unraveling the genetic diversity of Ceiba pubiflora (Malvaceae) in isolated limestone outcrops: Conservation strategies
Source: PLoS One. 2024 Apr 1;19(4):e0299361. doi: 10.1371/journal.pone.0299361 (PMC10984428; doi:10.1371/journal.pone.0299361)
Supplement: S5 Fig — (DOCX) [file pone.0299361.s005.docx]

**Unraveling the genetic diversity of *Ceiba pubiflora* (Malvaceae) in isolated limestone outcrops: conservation strategies**


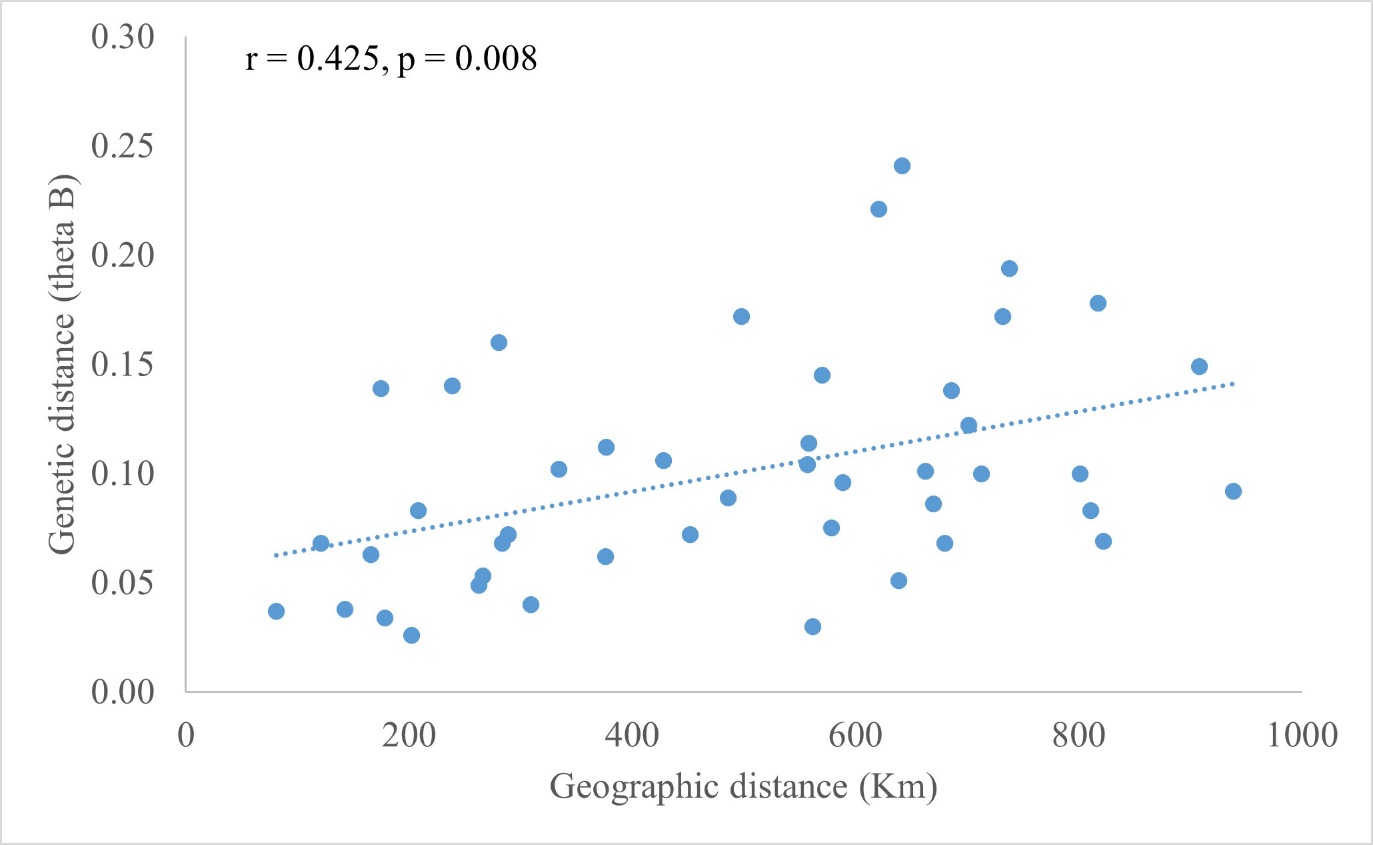


**S5 Fig. Relationship between geographic distances (Km) and genetic distance (Theta B) for *Ceiba pubiflora* populations**
