# Supplementary material for: Unraveling the genetic diversity of Ceiba pubiflora (Malvaceae) in isolated limestone outcrops: Conservation strategies
Source: PLoS One. 2024 Apr 1;19(4):e0299361. doi: 10.1371/journal.pone.0299361 (PMC10984428; doi:10.1371/journal.pone.0299361)
Supplement: S1 Table — The table presents the matrix of gene flow values (Nm) between all pairs of populations. (DOCX) [file pone.0299361.s006.docx]

**Unraveling the genetic diversity of *Ceiba pubiflora* (Malvaceae) in isolated limestone outcrops: conservation strategies**

**S1 Table. Historical gene flow contribution (%) among populations of *Ceiba pubiflora*.** The table presents the matrix of gene flow values (Nm) between all pairs of populations.

|  | BJL | MON | JAN | NOR | VIP | SAH | MAT | ARC | DOR | MIN |
| --- | --- | --- | --- | --- | --- | --- | --- | --- | --- | --- |
|  | 12% | 7.5% | 11% | 6.4% | 6.9% | 14.7% | 8.2% | 10.3% | 11.5% | 11.5% |
| BJL | - |  |  |  |  |  |  |  |  |  |
| MON | 7.71 | - |  |  |  |  |  |  |  |  |
| JAN | 9.33 | 7.20 | - |  |  |  |  |  |  |  |
| NOR | 4.04 | 2.74 | 4.73 | - |  |  |  |  |  |  |
| VIP | 9.50 | 2.58 | 5.68 | 6.70 | - |  |  |  |  |  |
| SAH | 16.69 | 4.26 | 12.84 | 6.48 | 5.16 | - |  |  |  |  |
| MAT | 3.69 | 3.01 | 6.83 | 4.63 | 3.21 | 12.91 | - |  |  |  |
| ARC | 5.29 | 4.49 | 4.00 | 2.04 | 1.71 | 10.02 | 3.19 | - |  |  |
| DOR | 6.52 | 5.29 | 4.47 | 2.34 | 1.83 | 6.98 | 5.67 | 13.23 | - |  |
| MIN | 4.48 | 4.58 | 7.01 | 2.78 | 2.39 | 7.68 | 3.14 | 14.25 | 18.73 | - |
